# Potential link between MHC–self-peptide presentation and hematopoiesis; the analysis of HLA-DR expression in CD34-positive cells and self-peptide presentation repertoires of MHC molecules associated with paroxysmal nocturnal hemoglobinuria

**DOI:** 10.1007/s12013-012-9435-1

**Published:** 2012-10-18

**Authors:** Jacek Nowak, Jolanta Wozniak, Ewa Mendek-Czajkowska, Agnieszka Dlugokecka, Renata Mika-Witkowska, Marta Rogatko-Koros, Elzbieta Graczyk-Pol, Anna Marosz-Rudnicka, Joanna Dziopa, Agnieszka Golec, Joanna Kopec-Szlezak, Krzysztof Warzocha

**Affiliations:** 1 Department of Immunogenetics Institute of Hematology and Transfusion Medicine, 14 Indira Gandhi Street, 02-776 Warsaw, Poland; 2 Laboratory of Immunophenotyping Institute of Hematology and Transfusion Medicine, Warsaw, Poland; 3 Department of Hematology Institute of Hematology and Transfusion Medicine, Warsaw, Poland

**Keywords:** Paroxysmal nocturnal hemoglobinuria, Hematopoietic stem cell, MHC molecules, HLA-DR expression, Self-peptide presentation, Regulatory T cells, Apoptosis, PNH clone domination, PNH clone selection

## Abstract

The mechanisms of MHC allele associations with paroxysmal nocturnal hemoglobinuria (PNH) and its aplastic anemia subtype (AA/PNH) remain unclear. It might be dependent on MHC molecule functional properties, such as a scope and frequency of antigen sampling and presentation. For documented PNH-associated MHC alleles we analyzed current reference databases on MHC molecule-eluted peptide presentation repertoires and searched for a range of presented peptides. MHC class II expression was measured on CD34+ cells and appeared to be increased in PNH patients. Two class I alleles (HLA-A*24:02 and B*18:01) have been previously confirmed to associate with protection and increased risk of AA/PNH, respectively. Their product molecules presented immunodominant epitopes derived from proapoptotic (serine/threonine–protein phosphatase) and antiapoptotic (phospholipase D), respectively, intracellular enzymes dependent on phosphoinositide (PI) content. For total PNH and non-aplastic PNH (n/PNH) subtype-associated DRB1*15:01 and DRB1*04:01 class II molecules presentation of exceptionally broad arrays of their own peptide fragments has been found. We conclude that self antigen peptides presented with high frequency in the context of MHC molecules of increased expression may be involved in the immune recognition and the regulation of HSC in the periphery. The block in the normal plasma membrane PI production due to the *PIG*-*A* mutation can help explain the differences in the activation of intracellular regulatory pathways observed between PNH and normal HSC. This is evident in the variation in MHC association patterns and peptide presentation repertoires between these two groups of patients.

**Electronic supplementary material:**

The online version of this article (doi:10.1007/s12013-012-9435-1) contains supplementary material, which is available to authorized users.

## Introduction

MHC molecules display extensive allelic variability and some of MHC alleles are known to associate with diseases of autoimmune pathogenesis. The main functional distinction of MHC molecules appears to be the scope and the frequency of peptide antigen sampling and presentation. Here we present an analysis that point to the role of MHC gene polymorphism and expression in the distinctive regulation of paroxysmal nocturnal hemoglobinuria (PNH) and normal hematopoietic stem cell (HSC) proliferation, that is reflected by MHC association pattern and peptide presentation repertoires.

Paroxysmal nocturnal hemoglobinuria (PNH) is a clonal nonmalignant disease caused by the somatic mutation of *phosphatidylinositol glycan complementation group-A (PIG*-*A)* gene located in chromosome Xp22.1 in hematopoietic progenitor cell [1]. The direct consequence of the mutation is the total or partial lack of the glycosyl phosphatidylinositol (GPI) [2] that can likely change the phosphatidylinositol (PI) turnover in plasma membranes.

The hemolysis, the main pathology in PNH is caused by the absence or deficiency of GPI-anchored proteins, such as decay accelerating factor (DAF) and membrane inhibitor of reactive lysis (MIRL) at plasma membranes of the mutated hematopoietic progenitor cell and all the progeny cells including mature erythrocytes [3–5]. The pathogenesis of other PNH-related disorders, like the bone marrow insufficiency, myelodysplasia and thrombosis is not yet clear.

The natural history of PNH is heterogeneous. It frequently follows the course with dominating hemolysis but can also be preceded by aplastic anemia or myelodysplastic syndrome and less frequently progress to, aplastic anemia or acute myeloid leukemia [6]. Forty percent of PNH patients develop venous thrombosis at some point in their illness [7]. This is the main cause of severe complications and death in PNH.

The GPI is necessary for anchoring of more than 20 membrane-bound proteins on the human blood cell surface [5]. This knowledge set the investigators’ faces for deficient functions of DAF, MIRL and other GPI-anchored membrane-bound proteins, but their lack alone was insufficient for PNH clonal dominance [8, 9]. Although this possibility has not yet been quantitatively tested it is noteworthy, when the biochemical pathway of GPI synthesis is blocked in PNH cells, this can alter the turnover rate for phosphoinositides (PI) in plasma membranes. There are at least two arguments for the altered PI in *PIG*-*A* mutant cells, such as (i) the biochemical balance, where the blocked biosynthesis of a product (i.e. GPI) is balanced with a substrate (i.e. PI) accumulation [1], and (ii) a pathway of PI delivery to plasma membranes, which is efficiently promoted by highly specific enzyme, the phosphatidylinositol transfer protein [10]. This transfer protein is able to deliver PI to plasma membranes at the expense of equimolar quantity of phosphatidylcholine (PC). In normal human granulocytes the molar concentration of PC exceeds PI by at least two orders of magnitude (data not published) and no pathways are known that can clear plasma membranes from excessive PI. Interestingly, the phosphorylation and dephosphorylation of PI are potent pathways functional in the regulation of the cell survival and apoptosis [11]. Provided altered PI in *PIG*-*A* mutant cells, the cell survival and apoptosis can be diversely regulated in PNH and normal HSCs.

Several models of PNH clone dominance were proposed by researchers involved in PNH studies. According to Luzzatto et al. [12] *PIG*-*A* mutation in HSC does not imply any harm as such. For PNH cone selection an additional element of bone marrow failure is necessary (dual pathogenesis model). Normal hematopoietic stem cells are eliminated via hypothetical molecule which, upon binding to a GPI-linked protein, can induce damage to the stem cell via immune mediated attack [13]. The nature of the GPI-linked protein and antigen molecule involved in this process remains unknown, so far. Young [14] proposed that within the hypocellular marrow environment, cytotoxic T lymphocytes were involved in the positive selection of the PNH clone. The key factor was the absence of GPI-anchored lymphocyte function-associated antigen-3 (LFA-3) on the PNH stem cells, an impaired ligation to T cell glycoprotein CD2 [15] and disrupted adhesion of cytotoxic T cells to target cells [16]. In this model both the immune-mediated bone marrow hypoplasia and the level of PNH clonality are determined by the relative balance of the T cell attack with LFA-3 loss [14]. However, it remains not clear why the increased cytotoxicity in numerous PNH patients is not suppressed by immunotherapy. In the three-step model proposed by Marsh and Elebute [17] the *PIG*-*A* mutation is followed by immunological attack to PIG-normal stem cells with limited clonal expansion caused by immunoselection. In this model a secondary mutation is a key factor of further expansion of PNH clone. The immunological attack is triggered by the unidentified PIG-anchored protein(s) and unknown second mutation is necessary for PNH clone domination [17]. Tiu and Maciejewski [18] proposed several possible mechanisms of PNH clonal evolution and expansion. Abnormal PNH cells could be a trigger for autoimmune process which, however, must have been more efficiently directed against normal hematopoietic cells. Alternatively, an initial immune attack would be directed against normal hematopoietic stem cells. The GPI-deficient hematopoietic stem cells are normally present as a small compartment in the bone marrow and can better survive the initial attack. The simultaneous growth advantage of PNH cells is explained by autoimmune-mediated pathway, as well. The proposed extrinsic mechanisms may include defective transduction of proapoptotic signals, dysfunction of proteasome, or other peptide processing pathway or disturbed lipid raft function due to lack of unidentified GPI-linked molecule(s). Hypothetical intrinsic mechanism of PNH hematopoietic stem cell growth advantage can involve additional mutation in regulatory genes leading to increased growth factor sensitivity, establishment of self-perpetuating autocrine loop or resistance to apoptosis [18]. However, the intrinsic growth advantage of PNH clone was impeached by Maciejewski et al. [19]. Although laboratory manifestations are mostly well established in these models of PNH clone dominance, the involvement of specific molecule or unique gene class across PNH patients is not well-documented and autoimmune trigger is unknown.

A role for the immune system in development of PNH has been supported by the finding of the association between PNH and certain allelic variants of MHC. In PNH patients increased frequencies of HLA-DR2 phenotype, DRB1*15:01 genotype and DRB1*15:01-DQA1*01:02-DQB1*06:02 haplotype have been regularly demonstrated [20–22]. Interestingly, the association of DQB1*06:02 alone (i.e. with the absence of DRB1*15:01) with PNH could not be confirmed because of complete linkage disequilibrium of these genes in populations [23]. In Italian PNH patients an increased frequency of the B*14:02-Cw*08:02 haplotype has been additionally suggested [22], indicating the role for MHC class I genes, at least in certain PNH subtypes. In our center, for the more uniform aplastic anemia PNH (AA/PNH) subtype the association with MHC class I B*18:01 allele has been recently revealed and for A*24:02 allele the protective role has been documented [24, 25].

Various mechanisms have been proposed to be responsible for MHC allele associations with diseases. Antiviral and antipathogen repertoires of the MHC molecules have mostly been exploited in this regard [26] but exact role of an infectious etiology in the pathogenesis of PNH remains elusive [22]. Apart from pathogen derived peptides, many peptide epitopes, fragments of normal (self) human proteins are presented in the context of MHC class I and II molecules with high efficiency [27]. It has been established in recent years that the autopeptide dependent regulatory T cell (T_reg_) mediated pathway is functional in central self-tolerance [28]. In this T_reg_-dependent pathway potentially autoreactive thymocytes are converted into regulatory T cells after cognate self tissue-specific peptide–MHC recognition on the thymic APCs. Self peptides derived from intracellular and tissue-specific proteins are displayed in the context of MHC molecules in a restrictive and hierarchical manner [28]. This data suggests that central deletion and anergy of self-specific T cells are not the only pathways of the self tolerance. The presentation of self peptides in the context of MHC molecules is actively used in immune cell cooperation. Moreover, the active self tolerance depends on the level of self antigen expression and reduced expression subverts the central tolerance process [29].

Hematopoiesis is regulated through multiple mechanisms, such as intercellular contacts of HSCs with components of bone marrow osteoblast niches, immunocompetent cells including T cells, and macrophages and through humoral components of growth factor/cytokine/hormone network [30–32]. Tissue-specific trigger for hematopoiesis suppression is not known. Of those pathways, the activity of GPI positive rather than GPI negative T cell subset and increased local production of IFN-γ are involved in mechanisms underlying altered hematopoiesis in PNH [33, 34]. Recently, it has been clearly established in transplantation and ectopic myelopoiesis models that contact-dependent interaction between CD4+CD25+FoxP3+regulatory T (T_reg_) cells and hematopoietic progenitor cells is required for the regulation of hematopoiesis and it is dependent on MHC class II [35, 36]. In early hematopoietic stem cells MHC class II molecules are highly expressed and their putative physiological role in regulation of hematopoiesis via T_reg_ TCR-peptide–MHC class II seems to be proven outside the immune and autoimmune response [35]. Considering extremely high specificity of TCR-peptide–MHC class II ligation this data suggest a crucial role of hematopoietic tissue-specific HSC-derived peptide epitopes and MHC expression level in regulation of hematopoiesis.

Therefore, we performed search analysis for the self-peptide presentation repertoires of PNH-associated HLA class I and II molecules including the analysis of source protein quality and functions. Simultaneously, we performed a pilot study of MHC class II expression on HSCs in PNH patients.

## Materials and methods

### Patients and controls

Seven PNH patients, five males and two females, aged 21–65 years, were enrolled in the study of HLA-DR expression in HSCs (Table 1). PNH diagnosis was documented by flow cytometry analysis of peripheral blood, using labeled monoclonal antibodies against GPI-linked CD59 and CD55 molecules, as described [24]. None of the enrolled patients was receiving any medical treatment along the study. Informed consent was obtained from individual patients before each blood sample collection. Five sex and age-matched healthy donors were used as controls.

**Table 1:** Characteristics of PNH patients at the disease diagnosis

Characteristics	PNH patients *N* = 7	
	Value	Range
Age (median, years)	30	21–65
G/CD59 (%)	88	53–98
G/CD55 (%)	89	43–94
Haptoglobin (g/L)	0	
LDH (U/L)	1106	239–1886
Platelets (×10^9^/L)	113	35–347
Leukocytes (×10^9^/L)	3.7	0.1–5.0
Reticulocytes (‰)	37	3–81
Erythrocytes (×10^12^/L)	2.91	2.19–3.80
Hemoglobin (g/dL)	8.0	6.5–9.7

Percent of CD59 and CD55 negative cells (PNH clone size) is presented; G, granulocyte fraction; CD, cluster of differentiation; LDH, lactate dehydrogenase

The clinical and laboratory characteristics of 50 PNH patients and 200 healthy controls enrolled to the association study and MHC allele associations have been described elsewhere [24]. Our previous results indicated the association of MHC class I A*24:02 (protection) and B*18:01 (susceptibility) alleles with AA/PNH subtype and the strong association of MHC class II DRB1*15:01 and DRB1*04:01 alleles and DRB1*15:01-DQB1*06:02 haplotype with both total PNH and non-aplastic PNH (n/PNH) subtype [24, 25].

### Cell preparation and flow cytometry

Specimens of 2 ml of EDTA whole peripheral blood were used for the analysis of HLA-DR expressing CD34-positive cells or GPI-anchored CD16 positive granulocytes. Cell surface markers were assessed by direct flow cytometry method using conjugated monoclonal antibodies against CD45 peridinin–chlorophyll–protein complex-cyanin 5.5 (PerCP-Cy5.5), clone 2D1; CD34 allophycocyanin (APC), clone 8G12; HLA-DR R-phycoerythrin (PE), clone L243 and CD16 APC-Cy7, clone 3G8 molecules (Becton–Dickinson, USA). Negative control was obtained by treating the cells with the isotype-matched control antibody (Becton–Dickinson, USA).

After staining (20 min, room temperature) and lysis the specimens were analyzed using FACSCanto flow cytometer with FACSDiva software (BD). At least 300 000 events were counted and data were recorded in the list mode. Detection of CD34+ cells was based on two-platform method ISHAGE protocol published by the International Society of Haematotherapy and Graft Engineering [37], designed as a set of guidelines for the accurate detection of CD34+ cells. The protocol was based on four-parameter flow cytometry method (CD45-PerCP/CD34-PE staining, and side and forward angle light scatter). Importantly, this approach allows the discrimination of HSCs (which express relatively low levels of CD45 on their surface) from lymphocytes and monocytes, and thus allowing the verification of “true” CD34+ cells as being dim for CD45 fluorescence and having low side scatter (CD45 dim, SSC low). To determine the HLA-DR expression on CD34 cells a triple labeling method with CD45/CD34/HLA-DR was used. To assess PIG positive granulocytes FSC/SSC, CD45/SSC and CD16 gating was applied. The density of surface HLA-DR was obtained by converting fluorescence intensity into the number of antigen molecules per cell, as measured by antibody-binding per cell (ABC). QuantiBRITE test (BD Biosciences, San Jose, CA) was applied for the measurement [38]. The mean channels of PE fluorescence were determined and ABC was than calculated with QuantiCALC software (BD Biosciences, San Jose, CA).

### Statistical Analysis and Comparisons

HLA-DR expression values in patients and controls were compared using two-tailed Student’s test for two samples with diverse variances. Correlation coefficient was determined by linear regression. Differences and correlations were considered significant for *P* < 0.05. Specificity of presented peptide repertoires was analyzed according to the putative functions of known source proteins and their potential roles in hematopoiesis and cell survival.

### Repertoire Analysis

Peptide presentation repertoires of HLA molecules have been extracted from Pub Med and MEDLINE database references and available repertoires of HLA class I and II restrictive elements associated with PNH (i.e., A*24:02, B*18:01, DRB1*04:01, and DRB1*15:01) have been selected. For comparison, available repertoires of HLA-A, B and DRB1 most frequent alleles, those non-associated with PNH (i.e., A*02:01, A*01:01, A*03:01, A*11:01, and A*25:01 with population frequencies [PFs] of 35, 25, 23, 13, and 12 %, respectively; B*07:02, B*08:01, B*51:01, B*15:01, B*27:05, B*35:01, and B*44:03 with PFs of 22, 17, 12, 9, 8, 8, and 8 %, respectively, and DRB1*07:01, DRB1*03:01, DRB1*01:01, DRB1*11:01, and DRB1*13:01 with PFs of 27, 22, 19, 13, and 10 %, respectively) have been selected (see Supplementary materials, Tables S1, S2, and S3).

In published data human cell lines of hematopoietic or β-cell origin were established including human EBV-transformed B-lymphoblastoid cell line or human pancreatic β-cell line. Detailed methods of human cell line establishment and sequencing of HLA-associated peptides were described elsewhere [39–44]. The ability to express of genes specifically active in hematopoiesis was verified by presentation of hemoglobin components in the context of some MHC molecules [45]. Only self peptides of human cell line origin those confirmed to be presentable by the direct elution from chromatographically purified HLA molecules and subsequent peptide sequencing have been included in analysis. Unlike the predicted peptides these peptides were considered presentable in the context MHC molecules on hematopoietic stem cells in vivo provided they were present in the proteomic content of the cell [46] and restrictive element was present. From a quantitative point of view, these MHC molecule-eluted peptides were more likely to belong to the pool of the immunodominant self peptides, whose qualitative distinction from non-dominant peptides is not yet characterized. Source proteins were identified according to Protein Information Resource Georgetown University Medical Center web page, edition Sep 2011 [47].

## Results

### Expression of HLA-DR on HSCs in PNH patients

**Fig. 1:**
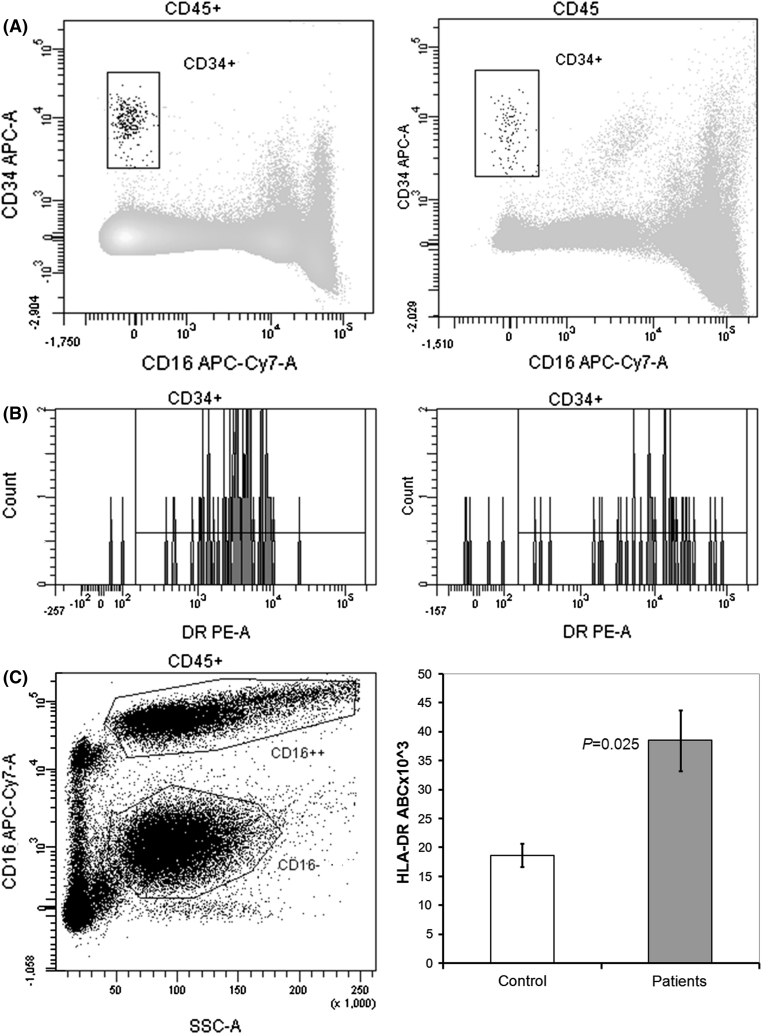
HSCs in PNH patients express increased numbers of HLA-DR molecules as compared to normal controls. **a** An example of HSC compartment (CD34-positive cells) in nucleated peripheral blood cells in control (*left*) and PNH patient (*right*). CD34-APC-positive cells were gated for on the CD45-PerCP-Cy-5.5/SSC plots. For better visualization of relatively small CD34 positive compartment CD16 positive granulocytes were subtracted from the pictures. **b** An example of HLA-DR staining in total CD34-positive cells in control (*left*) and PNH patient (*right*). Co-staining in the presence of CD45-PerCP-Cy-5.5, CD34-APC, and HLA-DR-PE mAb is shown. **c** An example of the CD16-APC-Cy7 GPI-anchored protein staining in granulocyte fraction (PNH clone size) in PNH patient (*left*) and results of seven experiments as median of HLA-DR antigen bound per CD34-positive cell (ABC) ± standard error of mean (*right*). APC, allophycocyanin; PE, R-phycoerithrine; PerCP-Cy-5.5, peridinin–chlorophyll–protein complex-cyanin 5.5; APC-Cy7, allophycocyanin-cyanin 7; HSCs, hematopoietic stem cells; PNH, paroxysmal nocturnal hemoglobinuria; mAb, monoclonal antibodies

Figure 1 shows an example of the results obtained by flow cytometry analysis in one representative PNH patient and control and collected HLA-DR expression in patient group. The HSC compartment (CD34-positive cells) was smaller in patient than in control (panels A right and left, respectively). HLA-DR expression on HSCs, as measured by antibody bound per cell (ABC) was higher in patient than in control (panel B right and left, respectively). PNH and normal clone sizes in the patient and the comparison of median HLA-DR in CD34-positive cells in total groups of patients and controls are also presented (panel C left and right, respectively).

The detailed flow cytometry results of patients are shown in Table 2. PNH clone size (CD16-positive cells) in PNH patients (median 60 %, range 48–99 %) was measured in granulocyte fraction assuming its best correlation with hematopoietic progenitor cell clone. Although median fraction of CD34-positive cells in peripheral blood nuclear cells was lower in PNH patients than in controls, the difference was not significant (median 0,014 vs. 0,034 % in patients and controls, respectively, *P* = 0.30). The compartments of circulating HSCs were more diverse in patients than in controls (range 0.08–0.70 % and 0.021–0.041 % in patients and controls, respectively) suggesting that levels of hypoplasia or phases of the disease were heterogeneous. As shown, the expression of HLA-DR molecules on CD34 positive peripheral blood cells was doubled in PNH patients as compared to controls when measured as ABC (median 38500 and 18600 HLA-DR molecules per cell in patients and control, respectively; *P* = 0.025), suggesting that the avidity of contact between HSCs with specific T cells can be elevated in PNH patients. Interestingly, the expression of HLA-DR in patient’s HSCs was not correlated with the PNH clone size (*r* = −0.35, *P* = 0.45), suggesting that the final HLA-DR expression on HSCs could be promoted by stimulating factors additional to PNH clone itself.

**Table 2:** Expression of HLA-DR molecules on CD34-positive peripheral blood cells and paroxysmal nocturnal hemoglobinuria (PNH) clone size in peripheral blood granulocytes in patients and controls

	Granulocytes	CD34	
ID	CD16 neg. (%)	Compartment (%)	HLA-DR ABC* ×10^3^
Controls			
C1	0	0.038	18.6
C2	0	0.034	13.5
C3	0	0.021	25.5
C4	0	0.032	16.5
C5	0	0.041	19.7
Patients			
P382	99.0	0.030	43.0
P404	60.0	0.019	46.1
P422	48.0	0.008	38.5
P461	48.0	0.011	51.9
P555	81.0	0.070	22.7
P605	94.0	0.014	16.3
P674	49.0	0.010	22.1
Median con.		0.034	18.6
Mean con.		0.033	18.8
±SE con.		0.003	2.0
Median pat.	60.0	0.014	38.5
Mean pat.	68.4	0.023	34.4
±SE pat.	8.5	0.008	5.2

* The difference between HLA-DR expression levels in CD34-positive cells in PNH patients and control was statistically significant (*P* = 0.025); CD34, circulating hematopoietic stem cells (HSCs); CD16 neg., PNH clone size (%); Compartment, fraction of peripheral blood HSCs (%); ABC, antibody bound per cell; HLA-DR ABC, number of HLA-DR antigen molecules per CD34-positive cell; SE, standard error of mean; pat., patients; con., controls

### Characteristics of peptide presentation repertoire and their source proteins

In presentation repertoire analysis peptides derived from several groups of functional proteins were displayed by PNH-associated HLA molecules (Table 3). Two product molecules encoded by A*24:02 and B*18:01 class I alleles presented peptides derived from proteins involved in cellular signaling. AA/PNH protective A*24:02 allele product presented the nonapeptide KYPENFFLL [39], an epitope derived from catalytic subunit of protein phosphatase (serine/threonine–protein phosphatase, EC 3.1.3.16) [47], the nonapeptide AYVHMVTHF [39] derived from testis enhanced gene transcript (BAX inhibitor 1) [47], the nonapeptide YYEEQHPEL [39] derived from interleukin (IL) 32 [47] and the nonapeptide VYIKHPVSL [39] from 26S proteasome non-ATPase regulatory subunit 8 [47]. The product of B*18:01 allele was associated with increased risk of AA/PNH and presented the oktapeptide GEDGRVYV [40] derived from phospholipase D (PI-PLD, phosphatidylinositol-glycan-specific phospholipase D, EC 3.1.4.50) [47], oktapeptide UEYARKUT [40] from 5-hydroxytryptamine 1F (5HT1F) receptor and nonapeptide DEKEKLQLV [40] from Hsp 47. The comparative analysis of peptide presentation repertoires indicated that MHC class I PNH-associated molecules have presented peptides with different amino acid motifs and generally different source proteins (Table 3) than those peptides presented by MHC class I PNH non-associated molecules (Supplementary materials, Table S1 and S2). Wide range of peptides was presented in the context of PNH non-associated MHC class I molecules. The peptides were derived from human proteins of different classes, including intracellular processing and trafficking associated proteins, enzymes, transcription factors, proteins related to apoptosis and cell cycle, structural proteins (e.g., hemoglobin components, ribosomal proteins) and HLA molecules themselves. Two PNH non-associated MHC class I molecules presented peptides derived from PI-PLD-dependent phosphatases and/or kinases (A*02:01 presented peptides from serine/threonine–protein phosphatase 2A and tyrosine protein kinase; B*27:05 presented peptides from phosphatidylinositol-3 kinase and serine/threonine–protein kinase, see Supplementary materials Tables S1 and S2). However, PI-PLD-derived peptides themselves were not presented in the context of PNH non-associated MHC class I.

**Table 3:** Summary of endogenous peptide presentation repertoires of HLA restrictive elements associated with PNH

Restrictive element	Repertoire**	Source protein	Amino acid positions	References
A*24:02	KYPENFFLL	Serine/threonine–protein phosphatase	91–99	[39]
A*24:02	AYVHMVTHF	Testis-enhanced gene transcript (BAX inhibitor 1)	45–53	[39]
A*24:02	YYEEQHPEL	Interleukin 32	107–115	[39]
A*24:02	VYIKHPVSL	26S proteasome non-ATPase regulatory subunit 8	224–231	[39]
B*18:01	GEDGRVYV	Phospholipase D (PLD)	753–760	[40]
B*18:01	UEYARKUT	5-hydroxytryptamine 1F receptor	142–150	[40]
B*18:01	DEKEKLQLV	Hsp 47	247–255	[40]
DRB1*04:01	VYPEVTVYPAK	DR4β (DRB1*04:01)	95–105	[43]
DRB1*04:01	VYPEVTVYPAKT	DR4β (DRB1*04:01)	95–106	[43]
DRB1*04:01	IYFRNQKGHSGLQPTGFLS	DR1β, DR4β, DR7β, DR15β	219–237	[44]
DRB1*04:01	IYFRNQKGHSGLQPTGLLS	DR53β (DRB4*01:01)	219–237	[44]
DRB1*04:01	IYFRNQKGSHSGLQPTGFL	Invariant DRβ (DRB1)	252–270	[48]
DRB1*04:01	SPEDFVYQFKGMCYF	DQ3.2β (DQB1*03:02)	3–17	[41]
DRB1*15:01	RVQPKVTVYPSKTQPLQH	DR15β (DRB1*15:01)	94–111	[41]
DRB1*15:01	RVQPKVTVYPSKTQP	DR53β (DRB4*01:01)	94–108	[41]
DRB1*15:01	DSDVGVYRAVTPQGRPDAEY	HLA-DQ6β, (DQB1*06:02)	41–60, 41–57, 41–58, 42–56, 43–58	[42]
DRB1*15:01	LEEFGRFASFEAQG	Invariant DRα (DRA)	45–58	[42]
DRB1*15:01	NIVIKRSNSTAATNEV	Invariant DQα (DQA)	97–112	[42]
DRB1*15:01	DVGVYRAVTPQGRP	Invariant DQβ (DQB)	43–57	[42]

** For peptide sequences one letter codes of amino acids were used

For PNH-associated MHC class II molecule presentation repertoires no peptides derived from proteins involved in cellular signaling or apoptosis were found (see Table 3). However, PNH-associated DRB1*15:01 and DRB1*04:01-encoded molecules displayed extremely wide ranges of peptides derived from MHC class II molecules (Table 3) as compared with presentation repertoires of other class II molecules (Supplementary materials Table S3). For DRB1*15:01-encoded molecule autopresentation of self 18-peptide RVQPKVTVYPSKTQPLQH derived directly from DRB1*15:01 molecule was accompanied by the presentation of DSDVGVYRAVTPQGRPDAEY 20-peptide and several of its shorter fragments derived from DQB1*06:02-encoded molecule [41]. Moreover, the same DRB1*15:01-encoded molecule presented also peptides LEEFGRFASFEAQG, NIVIKRSNSTAATNEV and DVGVYRAVTPQGRP derived from DRα, DQα, and DQβ MHC chain invariant sequences, respectively [42]. The DRB1*04:01 PNH-associated allele product displayed a set of peptides derived from itself and invariant DRB1 as well as from DQ3.2β molecule (Table 3). Both, DRB*15:01 and DRB*04:01 molecule presented fragments of tightly genetically linked haplotype component molecules, DQ6β and DR53β, respectively [42–44, 48]. The comparative analysis of peptide presentation repertoires indicated that MHC class II presented peptides with amino acid motifs typical for particular MHC molecule (Table 3). PNH-associated MHC class II alleles have presented peptides different than those peptides presented by PNH non-associated MHC class II most frequent alleles (see Supplementary materials Table S3). The peptides were derived from human proteins of different classes, including cellular receptors, enzymes, cell growth regulators, channel components and transporters, chaperone proteins, and HLA molecules themselves. Presentation of increased varieties of self MHC class II molecule fragments were found for PNH-associated MHC class II molecules.

Overall, peptide repertoires presented by MHC molecules seem to be components of those source proteins of high cellular turnover. They have the potential to form cell line-specific peptide signatures in vivo depending on currently degraded proteins [49]. After thymic selection and maturation of thymocytes [28] these self peptides can be specifically recognized by MHC class II-dependent regulatory T cells [35] and presumably, MHC class I-dependent CD8 positive naive T cells with TCR of low/intermediate affinity to MHC–peptide complexes [50].

## Discussion

The expression of type II GPI-anchored proteins is diminished or abolished in PNH clones [4, 51, 52]. Although GPI negative T cells produced lower levels of interferon gamma (IFN-γ), the overexpression of MHC in hematopoietic tissue is promoted by the increased levels of IFN-γ in GPI positive T cell subset [33, 34]. The diverse expression of MHC versus GPI-anchored proteins can shape the cell membrane characteristics and strongly influence interactions of the PNH clone with immune cells. As demonstrated, the HLA-DRB1*15:01 is restrictive element for the number of genetically “convinced” self peptides, i.e., those peptides endogenously derived from DRB1*15:01, the molecule encoded by its own restrictive allele, but also for the number of peptides encoded by genes tightly linked to DRB1*15:01 (i.e., DQB1*06:02, *DRA, DQA, DQB* genes) [41, 42]. Similar presentation pattern of broad range of DRB1*04:01 molecule itself and genetically ‘convinced’ peptides has been demonstrated for DRB1*04:01 molecule in human B cell lines, suggesting high presentability of these self peptides in HSCs. Comparatively, peptide fragments “convinced” by genetic linkage or belonging to invariant fragment of MHC were infrequently presented in the context of PNH non-associated DRB1 molecules and if presented the varieties of these peptides were narrow (Supplementary materials Table S3). The contribution of HLA-DR triggering in suppressed hematopoiesis was confirmed both in animal [53] and human model [54, 55]. We hypothesize that high expression of MHC in HSCs of PNH clone and the strong autologous signal made by the broad presentation of self class II peptides in DRB1*15:01 carriers can jointly effectively trigger T_reg_ suppressive activity directed at the level of MHC class II-expressing HSCs. After MHC class II-restricted activation the normal function of activated T_regs_ is MHC–peptide unrestricted contact with Th effector cells that are present in the vicinity, and have TCR of any antigenic specificity and IL-2 deprivation [56]. Consequently, both the bone marrow cellular and cytokine network are regulated toward growth suppression and apoptosis. Since, in PNH patients an increased percentage of IFN-γ producing cells was revealed [34] several apoptotic genes were stimulated in CD34-positive cells [57]. In the presence of excessive IFN-γ, the apoptosis is increased and the expression of MHC class I and II genes is enhanced thus boosting the link between MHC and PNH pathogenesis. In PNH patients increased MHC class II expression on HSCs was documented in this study. In normal individuals we found from 13,500 to 25,500 DRB1 molecules per hematopoietic stem cell. The experiment shows significantly higher expression of DRB1 molecules on unselected HSCs in PNH patients (median 38,500 DRB1 molecules per hematopoietic stem cell). In addition, in PNH patients the HLA-DR expression was independent on PNH clone size suggesting that finally both normal and *PIG*-*A* mutant cells can be involved in induction of MHC class II expression on HSCs.

The model of dominant presentation of certain self peptides fits well with our observation of higher association of DQB1*06:02-DRB1*15:01 haplotype than DRB1*15:01 with PNH [24]. The DQB1*06:02 allele in the tested population remains in complete linkage disequilibrium with DRB1*15:01, but the last allele is associated with several different DQB1 alleles [23]. In DRB1*15:01 carriers MHC self presentation by DRB1*15:01 molecule can be broader and triggering of T_reg_ activity can be more efficient in the presence of DQB1*06:02 allele than in DRB1*15:01 individuals with alternative DQB1 allele not presented in the context of DRB1*15:01 molecule. Our data suggest the dominant autopeptide presentation in the context of the MHC class II molecules and overexpression of MHC class II molecules in HSCs are common factors of T_reg_-mediated downregulation of hematopoiesis.

Papadopoulos et al. [40] have shown that the class I B*18:01 allele is specific restrictive element for immunodominating octapeptide of phosphatidylinositol-glycan-specific phospholipase D (PI-PLD) [40, 47]. Upon activation this intracellular enzyme has shown important anti-apoptotic and cell protective regulatory effect in hematopoietic cells [58, 59]. Simultaneously, phosphoinositides potentially disbalanced in *PIG*-*A* mutant PNH cells can be directly linked to the roles of PI-PLD in apoptosis [58], synthesis of phosphatidylinositol 4,5-bisphosphate (PIP2) [60] and the importance of the PIP2-specific PH domain in phospholipase D [61]. PIP2 is essential as a PI-PLD co-factor [61]. Evidence that PIP-kinase activity is autonomous can suggest that in the presence of higher concentrations of PI in PNH cells the PIP2 synthesis occurs with higher efficiency in *PIG*-*A* mutant PNH hematopoietic cells than in normal hematopoietic cells [60]. The PLD participate in Edg-3 receptor-mediated activation of the phosphatidylinositol 3-kinase (PI3K) and protein kinase B (PKB, also called Akt)-dependent pathway, which play a crucial role in the anti-apoptotic and pro-survival signaling [58]. Activation of the PKB/Akt leads to the inhibition of *Foxo* transcription factors, whose normal function is to drive the expression of cell death genes. This way metabolic block at the level of PI in PNH clone can activate PI-PLD and finally lead to the divergent sensitivity of normal and PNH clones to apoptosis.

*PIG*-*A*-deficient progenitors showed a gene expression signature similar to that seen in CD34-positive cells derived from healthy individuals, whereas “normal” CD34+ cells derived from PNH patients showed a pronounced expression of genes associated with apoptosis [62]. This data suggest that both normal and PNH HSCs are subjects of proapoptotic stress but the later cells are more resistant to apoptosis [63]. Relative resistance to apoptosis of PNH cells can be thus a consequence of increased PI-PLD activity in PIP2 enriched PNH cells [9, 64–66].

To explain why B*18:01 allele was significantly associated with increased risk of PNH and A*24:02 was protective we noticed that restricted fragments’ source protein enzyme activities are dependent on PI, dependent in turn on *PIG*-*A* gene mutation. It sounds reasonable the possibility that immunodominant presentation of the epitope of anti-apoptotic phosphatidylinositol-glycan-specific PLD can occur in the context of B*18:01 molecule provided high intracellular activity and high content of the enzyme protein [49]. Similarly, the immunodominant presentation of epitope derived from the serine/threonine protein phosphatase in the context of A*24:02 molecule is also feasible provided high intracellular content of protein phosphatase enzyme protein. The serine/threonine protein phosphatase is activated by PI3K (activated on the PLD pathway) and can disrupt the anti-apoptotic activity [58]. These enzymes (protein phosphatase and PI-PLD) have opposite function in apoptosis activation and both are dependent on PLD activated by PI. In accordance, it was established that activation of the anti-apoptotic human BCL2-related A1 and Mcl-1 proteins by PIP was associated with increased expression of related genes in PNH cells [64, 67, 68].

In contrast, a simple connection to different levels of phosphoinsitides in PNH and normal clones cannot be found for BAX inhibitor 1 [69], whose peptide epitope was presented in the context of A*24:02 molecule [39]. Consequently, overall anti-apoptotic activity of the BAX inhibitor 1 [69] cannot differentiate between PNH and normal clones. Consistently, similar expression of BAX gene in PNH and normal hematopoietic cells has been demonstrated by Horikawa et al. [65].

Although range of peptides presented in human cells can be broad and dependent mainly on peptide motif presentable by particular MHC [46], the TCR repertoire is restricted to thymic proteins and those non-thymic proteins specific for different tissues that are promiscuously expressed in thymic epithelial cells [28]. The putative function is the establishment of tissue-specific peptide signatures of low/intermediate affinity to TCRs, similar to these peptides of regulatory function in hematopoiesis [35]. PNH-associated MHC molecules can be particularly efficient presenters of certain peptide fragments of these promiscuous proteins naturally expressed in HSCs. MHC class II molecules themselves and PI-stimulated proteins can be specific for peripheral T_regs_ and naive CD8 positive T cells since their peptide fragments are presentable in the context of PNH-associated MHC molecules.

Frequent presentation of peptide fragments of active intracellular enzymes in the context of PNH-associated MHC class I molecules is likely driven by the proteome pool during the assembly of MHC class I molecules within endoplasmic reticulum (ER) [49]. The assembly process is known to be dependent on peptide availability. The frequent presence of selected peptide fragments within ER can affect following steps of external presentation and cell recognition. Consequently, the association of MHC class I molecules with AA/PNH occurrence can be dependent on the more frequent presence of cognate peptides inside the ER in PNH HSCs than in normal HSCs. Higher expressions of MHC class I molecules in HSCs of PNH patients than in normal bone marrow can increase overall avidity of cell recognition. To assess MHC class I expression in PNH cells Nagakura et al. [70] have examined *PIG*-*A* gene-transfected *PIG*-*A* deficient cells in vitro and compared with *PIG*-*A* mutant untransfected cells. They found comparable MHC class I expressions in *PIG*-*A* positive and negative cell cultures. However, this result can be biased by the lack of feedback from INFγ producing bone marrow cells, which is expected in PNH patients in vivo [34].

Increased AA/PNH in patients with MHC molecules presenting PI-dependent enzyme peptides can be explained by what is currently known about central thymic and peripheral selection of T cells [50, 71]. Thymocyte apoptosis and central deletion is directed to TCR expressing cells with high affinity for MHC–self-peptide complexes, whereas self-reactive T cells with intermediate/low affinity for self antigen escape thymic negative selection. Only those intermediate/low affinity TCR bearing cells are released into the periphery forming naive T cell compartment. If the avidity of HSC/CD8 positive naive T cell contact is variable it can be increased close to the activation threshold jointly by dominant presentation of certain self peptides and elevated expression of MHC class I molecules. Further maturation of those hypo-activated CD8+ T cells can direct limited cytotoxicity toward PNH and normal HSCs, those presenting line-specific peptides. Aplastic anemia picture in some PNH patients and the association of certain MHC class I with AA/PNH subtype can be explained by this process.

### Running Hypothesis

Our study is in agreement with earlier findings that the mechanisms of the clonal selection and functional pathologies of the PNH hematopoietic cells are complex and involve immunorecognition and apoptosis. Downregulation of the hematopoiesis rather than cytotoxicity is the effect of MHC class II-dependent immunorecognition because of intermediate/low affinity of MHC–self peptide-TCR ligation. The emerging picture for the domination of the PNH clone over normal HSC clones is that the self peptide repertoires are diversely presented by HSC located MHC class II to T_regs_ and anti-apoptotic pathways are diversely modulated by PI content in *PIG*-*A* mutant and normal HSCs.

**Fig. 2:**
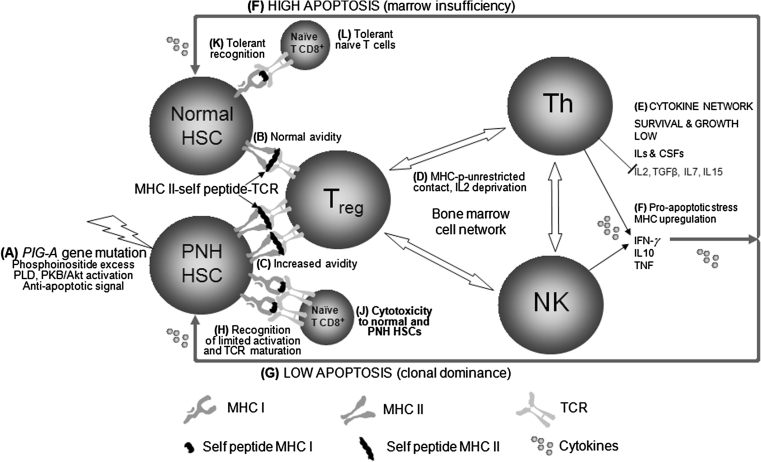
Hypothetical pathways involved in MHC–self peptide-TCR recognition and PNH clone dominance. Both immunorecognition and apoptosis mechanisms are involved in clonal selection of PNH hematopoietic cells. Down-regulation of hematopoiesis and limited cytotoxicity are direct effects of low affinity MHC–self peptide-TCR immunorecognition. Self peptide repertoires are diversely presented by HSC-located MHC class II to T_regs_ and apoptotic pathways are diversely modulated by PI content in *PIG*-*A* mutant and normal HSCs. (*A*) A somatic mutation occurs in *PIG*-*A* gene in a hematopoietic stem cell followed by gradual PI imbalance in plasma membranes, followed by putative PLD, PI3K, and PKB/Akt activation and suppression of *Foxo* transcription factors. This makes the *PIG*-*A* mutant HSCs more resistant to apoptosis than normal HSCs. (*B*) T_reg_-mediated self recognition of HSCs (including complex MHC class II-self peptide) results in the T_reg_-activation, MHC unrestricted intercellular contact with Th CD4+ cells of any antigenic specificity and possibly other effector cells that are present in the bone marrow in the vicinity of activated T_regs_. (*C*) The induction of this process is more productive if increased avidity of the recognition is the case, i.e., when more efficient presentation of certain HSC-derived self peptides occurs in the context of PNH associated than non-associated MHC class II molecules. (*D*) The bone marrow “cellular network” is downregulated, that results in IL-2 deprivation, and (*E*) the “cytokine network” shift toward IFN-γ production, leading to the “pro-apoptotic stress” and MHC class I and II up-regulation. The bone marrow turns to the picture of bone marrow insufficiency. In local bone marrow hematopoietic foci PNH cells overcome the pro-apoptotic stress by the *PIG*-*A* gene mutation, PI alteration and activation of anti-apoptotic pathway, so that (*F*) high and (*G*) low apoptosis, respectively, occurs in normal and PNH HSCs. At this stage the anti-apoptotic loop is closed between IFN-γ-producing cells and *PIG*-*A* mutant hematopoietic stem cells creating the mechanism of PNH clone domination. (*H*) Excessive presentation of certain HSC-derived self peptides in the context of up-regulated MHC class I can increase avidity of specific TCR of naive CD8-positive T cells close to the activation threshold and induce maturation of TCR. (*J*) Normal and PNH HSCs presenting line-specific peptides are attacked by these mature “post-naive” T cells and aplastic anemia picture is presented. This process is triggered by more efficient presentation of certain HSC-derived self peptides in the context of PNH associated than non-associated MHC class I. (*K*) Normal HSCs present normal levels of tissue-specific peptides. (*L*) Recognition with normal MHC–self peptide-TCR avidity is tolerant. HSC, hematopoietic stem cell; MHC, mayor histocompatibility complex; T_reg_, regulatory T cell; naïve T CD8+, naive cytotoxic T cell; Th, helper T cells; NK, natural killer cells; TCR, T cell receptor; IL, interleukin; CSF, colony stimulating factor, TGFβ, transforming growth factor beta; IFN-γ, interferon gamma; TNF, tumor necrosis factor

Taken together these data on intracellular metabolic pathways, intercellular recognition patterns, and the role of T_regs_ in regulation of hematopoiesis in accordance with our data on MHC expression add new to our current understanding of normal and PNH type hematopoiesis. A running hypothesis can be formulated on the domination of the PNH clone over normal HSC clones (see Fig. 2). First, a somatic mutation occurs in *PIG*-*A* gene in hematopoietic stem cell followed by gradual PI imbalance, direct PI-PLD, and subsequent PI3K and PKB/Akt activation and suppression of *Foxo* transcription factors. This makes the *PIG*-*A* mutant HSCs more resistant to apoptosis than normal HSCs. Second, increased T_reg_-mediated self recognition of HSCs (including MHC class II-self peptide complex) results in the T_reg_-activation, intercellular contact with Th CD4+ cells of any antigenic specificity and possibly other effector cells that are present in the bone marrow in the vicinity of activated T_regs_. Certain MHC class II molecule fragments are efficiently presented in the context of PNH-associated MHC class II molecules themselves and more efficiently trigger T_reg_-mediated self recognition. At the third stage, the bone marrow “cellular network” is downregulated, that results in IL-2 deprivation and the “cytokine network” shift toward IFN-γ production, leading to both the “pro-apoptotic stress” and MHC class I and II upregulation on PNH and normal HSCs. The bone marrow turns to the picture of bone marrow insufficiency. In local bone marrow hematopoietic foci PNH cells are “well prepared” to overcome the pro-apoptotic stress by the *PIG*-*A* gene mutation, PI imbalance and activation of antiapoptotic pathway, so that high and low apoptosis occurs in normal and PNH HSCs, respectively. At this stage the antiapoptotic loop is closed between IFN-γ-producing cells and *PIG*-*A* mutant hematopoietic stem cells creating the mechanism of PNH clone domination. Excessive presentation of certain HSC-derived self peptides in the context of upregulated MHC class I can create kind of immunodominance and increase the avidity of specific TCR on naive CD8-positive T cells close to the activation threshold. Consequently, TCR maturation and cytotoxicity toward the total HSC line is generated by the activated naive CD8-positive T cells. This process can be triggered more efficiently by the presentation of certain HSC-derived self peptides in the context of PNH associated than non-associated MHC class I. In this model MHC molecule self peptide presentation repertoires make the difference between PNH-associated, and non-associated MHC and MHC expression differentiates between PNH and normal hematopoiesis creating the framework for MHC association with PNH.

### Rationale

It was already noticed by Luzzatto et al. [12] that downregulation of apoptotic pathways can result, in the more advantageous PNH-type hematopoiesis in otherwise aplastic anemia-type hematopoiesis. However, in the previously proposed models [12, 17, 18] a selective element of MHC-restricted cytotoxicity toward *PIG*-normal cells is still sought and second mutation is not an uniform feature of *PIG*-*A* mutant clone. Current lack of data on which hematopoietic tissue-specific peptides are active in self recognition in vivo and unknown levels of PI in PNH hematopoietic progenitor cells are weak points of the presented hypothesis. However, a number of still open points in current understanding of clonal dominance in PNH can be better understood. An additional element of bone marrow failure in dual pathogenesis model of PNH clone dominance proposed by Luzzatto et al. [12, 13] and a hypocellular marrow environment element proposed by Young et al. [14] can be justified by excessive T_reg_ activation and local induction of IFN-γ. A role of cytotoxic T lymphocytes in Young’s model [14], an immunoselection proposed by Marsh and Elebute [17] and aplastic anemia picture observed in some PNH patients can be legitimated by activation of self specific naive CD8-positive T cells with more avid TCR. Finally, defective transduction of proapoptotic signals proposed by Tiu and Maciejewski [18] can be explained by the metabolic block and activation of antiapoptotic phosphoinositide second messengers in *PIG*-*A* mutant cells.

During our analysis we put forward several basic questions on cellular recognition, such as why HSCs physiologically express the MHC class II, why MHC molecules physiologically present self peptides, why MHC class II molecules present ‘convinced’ self peptides (derived from themselves or proteins encoded by tightly genetically linked genes) and why MHC class I molecules present peptide fragments of vitally important intracellular pathways? Our hypothesis addresses these questions and consistent answer is proposed that these features are set at the cellular recognition level for the active and strict regulation of hematopoiesis.

Future study on membrane PI turnover, tissue-specific T cell recognition, and TCR maturation, and avidity of T_reg_ and naive CD8-positive TCR will elucidate hematopoietic stem cell biology and show novel therapeutic strategies in PNH. Further investigations in these interesting areas are warranted.

## Electronic supplementary material

Below is the link to the electronic supplementary material.

Supplementary material 1 (DOC 394 kb)
