# Supplementary material for: Potential link between MHC–self-peptide presentation and hematopoiesis; the analysis of HLA-DR expression in CD34-positive cells and self-peptide presentation repertoires of MHC molecules associated with paroxysmal nocturnal hemoglobinuria
Source: Cell Biochem Biophys. 2012 Oct 18;65(3):321–33. doi: 10.1007/s12013-012-9435-1 (PMC3601265; doi:10.1007/s12013-012-9435-1)
Supplement: Supplementary file 1 — Supplementary material 1 (DOC 394 kb) [file 12013_2012_9435_MOESM1_ESM.doc]

##### Supplementary materials

##### Tables and legends

Table S1. Summary of endogenous peptide presentation repertoire source proteins of HLA A restrictive elements non-associated with PNH. Repertoires of 5 most frequent molecules encoded in A locus are presented. Population frequencies of A*02:01, A*01:01, A*03:01, A*11:01 and A*25:01 molecules were 35%, 25%, 23%, 13% and 12%, respectively [1]. For peptide sequences one letter amino acid codes were used. X – any amino acid.

| **Restrictive element** | **Repertoire** | **Source protein** | **Amino acid positions** | **Reference** |
| --- | --- | --- | --- | --- |
| A*02:01 | TLWVDPYEV | B-cell translocation gene 1 protein | 103-111 | [2,3,4,5] |
|  | FLLDHLKRV | Breakpoint cluster region protein | 1181-1189 | [6] |
|  | VLFRGGPRGLLAV | Translocon-associated protein subunit alpha | 19-31 | [4] |
|  | VLFRGGPRGLLAVA | Translocon-associated protein subunit alpha | 19-32 | [3] |
|  | SLLPAIVEL | Serine/threonine-protein phosphatase 2A | 370-378 | [2,5] |
|  | YLLPAIVHI | ATP-dependent RNA helicase p68 | 225-233 | [2,5] |
|  | FLLPTGAEA | Cathepsin G | 10-18 | [6] |
|  | LLDPKLUYLL | CD3 ζ chain | 17-24 | [6] |
|  | SLPHFHHPET | Erythrocyte membrane protein band 4.9, dematin | 124-133 | [6] |
|  | MLLSVPLLLG | Calreticulin | 1-10 | [3] |
|  | VLSPADKTNVK | Hemoglobin α 1 | 2-12 | [6] |
|  | LLYDMVUGDIP | Serine/threonine-protein kinase pim-3 | 234-244 | [6] |
|  | LLDVPTAAV | Gamma-interferon-inducible lysosomal thiol reductase IP-30 | 16-24 | [2,3,4,5] |
|  | LLLDVPTAAV | Gamma-interferon-inducible lysosomal thiol reductase IP-30 | 15-24 | [3] |
|  | LLLDVPTAAVQ | Gamma-interferon-inducible lysosomal thiol reductase IP-30 | 15-25 | [4] |
|  | LLLDVPTAAVQA | Gamma-interferon-inducible lysosomal thiol reductase IP-30 | 15-26 | [3] |
|  | EXVDXXEKV | Uncharacterized |  | [2] |
|  | SXPSGGXGV | Uncharacterized |  | [2,5] |
|  | GXVPFXVSV | Uncharacterized |  | [2,5] |
|  | SXXVRAXEV | Uncharacterized |  | [2,5] |
|  | VXXPKXXXX | Uncharacterized |  | [2] |
|  | KXNEPVXXX | Uncharacterized |  | [2,5] |
|  | MVDGTLLLL | HLA-E signal sequence | 1-9 | [6] |
|  | UIALFALPF | Interferon α/β receptor α chain | 376-383 | [6] |
|  | YMAPEILMRS | Ribosomal S6 kinase | 236-245 | [6] |
|  | IPRAEVAELL | Tyrosine-protein kinase Fes/Fps | 465-474 | [6] |
|  | FIYNADLMNU | GM CSF receptor α chain | 127-135 | [6] |
|  | KVNVDEVGGE | Globin β chain | 18-27 | [6] |
|  | KQYESVLMVSI | IL-7 | 32-42 | [6] |
|  |  |  |  |  |
| A*01:01 | STDHIPILY | Fructose-6-amino transferase | 217-225 | [7] |
|  | ATDFKFAMY | Cyclin D | 135-143 | [7] |
|  | GTDEXRNXY | Uncharacterized |  | [7] |
|  | DSDGSFFLY | Ig γ-4 chain | 279-287 | [7] |
|  | YLDDPDLKY | Cytosine methyl transferase | 238-246 | [7] |
|  | IADMGHLKY | Proliferation cell nuclear antigen | 241-249 | [7] |
|  | VADKVHXMY | E3 ubiquitin/ISG15 ligase TRIM25 | 593 - 601 | [7] |
|  | FTDVNSXXRY | Bifunctional glutamate/proline tRNA ligase | 48 - 57 | [7] |
|  | ETDXXXDRSEY | Uncharacterized |  | [7] |
|  | YTDYGGLIFNSY | Cytochrome C oxidase II |  | [7] |
|  | VSDIVGPDGLVY | Fibrillarin | 177-188 | [8] |
|  | SSEQTFMY | Ornithine decarboxylase | 309-317 | [8] |
|  | STEPVNILY | Homo sapiens nuclear protein in testis (NUT) | 899-904 | [8] |
|  | FTEVSIRKY | Tyrosine-protein kinase Mer | 112-117 | [8] |
|  | MIEPRTLQY | Ribosomal protein S16 | 41-49 | [8] |
|  | ITEDMGHLKY | Transcription factor Ovo-like 1 | 62-67 | [8] |
|  | YTSDYFISY | Ets-1 | 154-162 | [7] |
|  | YTAVVPLVY | Ig J chain | 102-110 | [7] |
|  | YTNPQFNVY | RAS p21 protein activator 2 | 218-222 | [7] |
|  | ETXXPDWSY | Uncharacterized |  | [7] |
|  |  |  |  |  |
| A*03:01 | RTQNVLGEK | Ribosomal protein S3 | 54-63 | [7] |
|  | SVLNLVIVK | Ribosomal protein S6 | 107-115 | [7] |
|  | SVLNLVIVK | Apoptosis related protein PNAS-20 | 36-44 | [7] |
|  | ASFDKAKLK | Thymosin β-10 | 11-19 | [7] |
|  | KVVNPLFEK | Ribosomal protein L7a | 26-34 | [7] |
|  | AAMLDTVVFK | 26S proteasome non-ATPase regulatory subunit 14 | 301-310 | [7] |
|  | GTMTTSXYK | Uncharacterized |  | [7] |
|  | RVEQAVESMVK | Protein FAM136A | 8-18 | [7] |
|  |  |  |  |  |
| A*11:01 | SVLNLVIVK | Ribosomal protein S6 | 107-115 | [7] |
|  | SVLNLVIVK | Apoptosis related protein PNAS-20 | 36-44 | [7] |
|  | KVVNPLFEK | Ribosomal protein L7a | 26-34 | [7] |
|  | GTMTTSXYK | Uncharacterized |  | [7] |
|  | RTQNVLGEK | Ribosomal protein S3 | 54-63 | [7] |
|  | GQYGNPLNK | Bovine metalloproteinase | 19-27 | [9] |
|  | ASFDKAKLK | Thymosin β-10 | 11-19 | [7] |
|  | ASFDKAKLKK | Thymosin β-10 | 11-20 | [9] |
|  | AAMLDTVVFK | 26S proteasome non-ATPase regulatory subunit 14 | 301-310 | [7] |
|  | AVMKPEAEKRK | Uncharacterized |  | [9] |
|  | RVQEAVESMVK | Protein FAM136A | 8-18 | [7] |
|  | YFDPANGKFSK | Elongation factor 2 | 265-275 | [9] |
|  | AVILPPLSPYFK | NADH dehydrogenase (Ubiquinone) 1 alpha subcomplex | 29-40 | [7] |
|  | ATAGDGLIELRK | Prohibitin | 229-240 | [7,9] |
|  | GVMPSHFSR | Ribosomal protein S19 | 93-101 | [9] |
|  | STYYGSFVTR | Eukaryotic translation initiation factor 3 | 136-145 | [9] |
|  |  |  |  |  |
| A*25:01 | ETINEEAAEW | HIV-1 p24 gag | 203-212 | [10] |
|  | VMSNTLLSAW | EBV LMP2A | 442-451 | [11] |

Table S2. Summary of endogenous peptide presentation repertoire source proteins of HLA B restrictive elements non-associated with PNH. Repertoires of 7 most frequent molecules encoded in B locus are presented. Population frequencies of B*07:02, B*08:01, B*51:01, B*15:01, B*27:05, B*35:01 and B*44:03 molecules were 22%, 17%, 12%, 9%, 8%, 8% and 8%, respectively [1]. For peptide sequences one letter amino acid codes were used. X – any amino acid.

| **Restrictive element** | **Repertoire** | **Source protein** | **Amino acid positions** | **Reference** |
| --- | --- | --- | --- | --- |
| B*07:02 | APRASRPSL | Uncharacterized |  | [12] |
|  | APRTLVLLL | HLA-A*0201 signal sequence | 5-13 | [13] |
|  | SPRYIFTML | Topoisomerase II | 801-809 | [13] |
|  | RPKSNIVLL | CD20 | 222-230 | [12,13] |
|  | APRXPXTGX | Uncharacterized |  | [13] |
|  | APRASRPSX | Uncharacterized |  | [13] |
|  | APRAXXXXX | HLA-B13, B15 and B57 | 46-49 | [13] |
|  | APRSNGMVX | Uncharacterized |  | [14] |
|  | APAPTVAVX | Uncharacterized |  | [13] |
|  | APYGGPIAL | Vacuolar protein sorting-associated protein 16 | 41-49 | [14] |
|  | MPRGVVVTX | E3 ubiquitin-protein ligase HECTD1 | 851-859 | [13] |
|  | RPSGPGPEL | Actin dependent regulator of chromatin SMARCA2 | 261-268 | [13] |
|  | APRTVALTA | HLA-DP signal sequence | 9-17 | [12,13] |
|  | APRQPGLMA | Coiled-coil-helix-coiled-coil-helix domain-containing protein 2 | 49-54 | [14] |
|  | RPRHQGVMV | β Actin | 37-45 | [12] |
|  | APRPPPKPM | Ribosomal protein S26 | 107-115 | [14] |
|  | APRTVALTAL | HLA-DP intracellular domain | 9-18 | [13] |
|  | APRAFXPXPV | Uncharacterized |  | [13] |
|  | LVMAPRTVL | HLA-B*0702 intracellular domain | 2-10 | [13] |
|  | RVMAPRALL | HLA-C*0701, C*0702, C*18 intracellular domain | 2-10 | [12] |
|  | RVMAPRAXX | HLA-C*0701, C*0702, C*18 intracellular domain | 2-10 | [13] |
|  | AASKERSGVSL | Histone H1 | 49-59 | [12,13] |
|  |  |  |  |  |
| B*08:01 | HPKYKTEL | Tristetraproline | 148-155 | [8] |
|  | EPKYKTQL | Phosphoribosyl aminoimidazole succinocarboxamine synthase | 95-102 | [8] |
|  |  |  |  |  |
| B*51:01 | DAHIYLNHI | Thymidylate synthase | 253-261 | [15] |
|  | YPFKPPKV | Homology to yeast UBC5 | 61-68 | [15] |
|  | IPPEVNRQL | Uncharacterized |  | [15] |
|  | TGYLNTVTV | Guanine nucleotide-binding protein, GBLP | 192-200 | [15] |
|  |  |  |  |  |
| B*15:01 | SQFGGGSQY | Eukaryotic translation initiation factor 3 subunit D | 61-69 | [16,17] |
|  | GQRKPATSY | Homology to rat ribosomal protein L28 | 68-76 | [16] |
|  | IQPGRGFVLY | DNA-directed RNA polymerase II subunit RPB7 | 59-68 | [16] |
|  | GQRKGAGSVF | Ribosomal protein L8 | 7-16 | [17,18] |
|  | VQGPVGL | Collagen α1 | 1106-1112 | [16] |
|  | GQRKGAGSV | Homology to rat ribosomal protein L8 | 7-15 | [16] |
|  | YLGEFSITY | Ribosomal protein S15 | 114-122 | [16] |
|  | KIKSFVKVY | Ribosomal protein L27 | 66-74 | [16] |
|  | VLKPGMVVTF | Elongation factor 1 α | 271-280 | [16] |
|  | IAVGYV | HLA class I heavy chain | 23-28 | [18] |
|  |  |  |  |  |
| B*27:05 | ARLQTALL | Homology to rat core histone | 188-196 | [19] |
|  | RRFTRPEH | Ubiquitin carboxyl-terminal hydrolase 22 | 367-374 | [20] |
|  | RRSKEITVR | ATP-dependent RNA helicase | 77-85 | [20] |
|  | KRFEGLTQR | Serine/threonine-protein kinase 38-like | 444-452 | [20] |
|  | HRAQVIYTR | 40S ribosomal protein S25 | 103-111 | [21] |
|  | FRYNGLIHR | Homology to rat 60S ribosomal protein L28 | 37-45 | [20] |
|  | RRIKEIVKK | HSP89 α | 201-209 | [20] |
|  | PRVKEVVKK | HSP89 β | 195-203 | [20] |
|  | GRIDKPILK | Homology to yeast/slime mould ribosomal proteine | 173-181 | [20] |
|  | GRFEGTSTK | Neuronal acetylcholine receptor | 141-149 | [21] |
|  | ARLFGIRAK | 60S ribosomal protein L13 | 189-197 | [19,20] |
|  | RRISGVDRY | NADH dehydrogenase (Ubiquinone) 1 alpha subcomplex | 52-60 | [20] |
|  | RRFFPYYVY | Proteasome subunit C5 | 127-135 | [22] |
|  | PRVLVQVSY | Methionine adenosyltransferase | 312-320 | [21] |
|  | RRFGDKLNF | Immediate-early response gene | 87-95 | [19] |
|  | KRFSFKKSF | Homology to bovine myristoilated alanine-rich C-kinase substrate | 155-163 | [19] |
|  | GRLTKHTKF | Homology to rat ribosomal protein L36 | 36-44 | [19,22] |
|  | GRFGSGMNM | hnRNA-binding protein M4 | 360-368 | [21] |
|  | GRTFIQPNM | Amidophosphoribosyltransferase precursor | 354-362 | [21] |
|  | LRFQSSAVM | Histone | 83-91 | [21] |
|  | RRLPIFSRL | TIS 11B protein | 325-333 | [19] |
|  | RRYQKSTEL | Histone H3.3 | 52-60 | [20] |
|  | TRYPILAGH | Cytochrome P450 | 20-28 | [19] |
|  | RRWLPAGDA | Elongation factor 2 | 341-349 | [20] |
|  | RRYDRKQSGY | 60S ribosomal protein L44 | 39-48 | [21] |
|  | GRFNGQFKTY | Ribosomal protein S21 | 44-53 | [22] |
|  | GRKTGQAPGY | Cytochrome C | 38-47 | [21] |
|  | GRWPGSSLYY | Lamin B receptor | 14-23 | [21] |
|  | KRWQAIYKQF | Ca²+-dependent protease | 172-181 | [21] |
|  | GRILSGVVTK | 40S ribosomal protein S11 | 70-79 | [21] |
|  | RRIKEIVKKH | HSP 86 | 200-209 | [19] |
|  | RRYLENGKETL | HLA class I heavy chain | 169-179 | [22] |
|  | RRMGPPVGGHR | Ribonucleoprotein L | 312-322 | [19] |
|  | RRFVNVVPTFGK | 40S ribosomal protein S30 | 114-125 | [21] |
|  | RKGGNNKLIK | Phosphatidylinositol-3 kinase | 373-382 | [21] |
|  | UULNSQDQQCDSSLVE | Homology to DRAF-1 *Drosophila* protooncogene | 1-16 | [23] |
|  |  |  |  |  |
| B*35:01 | LPFDFTPGY | Uncharacterized |  | [24] |
|  | LPGPKFLQY | Uncharacterized |  | [24] |
|  |  |  |  |  |
| B*44:03 | SEIDLILGY | Uncharacterized |  | [25] |
|  | SEIDTVAKY | Uncharacterized |  | [25] |
|  | AEIPTRVNY | Uncharacterized |  | [25] |
|  | AEIPRTFKY | Uncharacterized |  | [25] |
|  | DEVGIVTKY | Uncharacterized |  | [25] |
|  | AEMGKGSFKY | Elongation factor 2 | 48-57 | [26] |
|  | DEVGIVTKMY | Uncharacterized |  | [25] |
|  | AEDKENYKKF | HSP86 | 420-429 | [26] |
|  | AEDKENYKKF | HSP84 | 428-437 | [26] |

Table S3. Summary of endogenous peptide presentation repertoire source proteins of HLA DRB1 restrictive elements non-associated with PNH. Repertoires of 5 most frequent molecules encoded in each locus are presented. Population frequencies of DRB1*07:01, DRB1*03:01, DRB1*01:01, DRB1*11:01 and DRB1*13:01 molecules were 27%, 22%, 19%, 13% and 10%, respectively [1]. For peptide sequences one letter amino acid codes were used. X – any amino acid.

| **Restrictive element** | **Repertoire** | **Source protein** | **Amino acid positions** | **Reference** |
| --- | --- | --- | --- | --- |
| DRB1*07:01 | RPAGDGTFQKWASVVVPSGQ | HLA-A29 | 234-253 | [27] |
|  | RPAGDGTFQKWASVVV | HLA-A29 | 234-249 | [27] |
|  | GDGTFQKWASVVVPSGQEQRYT | HLA-A29 | 237-258 | [27] |
|  | GDGTFQKWASVVVPSGQE | HLA-A29 | 237-254 | [27] |
|  | GTFQKWASVVVPSG | HLA-A29 | 239-252 | [27] |
|  | GTFQKWASVVVPSGQ | HLA-A29 | 239-253 | [27] |
|  | GTFQKWASVVVPSGQEQRYTCHV | HLA-A29 | 239-261 | [27] |
|  | RETQISKTNTQTYRENL | HLA-B44 | 62-78 | [27] |
|  | RETQISKTNTQTYREN | HLA-B44 | 62-77 | [27] |
|  | RETQISKTNTQTYRE | HLA-B44 | 62-76 | [27] |
|  | RSNYTPITNPPEVTVLTNSPVELREP | HLA-DR α chain | 101-126 | [27] |
|  | GALANIAVDKANLEIMTKRSN | HLA-DR α chain | 58-78 | [27] |
|  | SLQSPITVEWRAQSESAQSKMLSGIGGFVL.. | HLA-DQ α chain | 179-208 | [27] |
|  | VTQYLNATGNRWCSWSLSQAR | 4F2 | 318-338 | [27] |
|  | VTQYLNATGNRWCSWSL | 4F2 | 318-334 | [27] |
|  | GDMYPKTWSGMLVGALCALAGVLTI | K+ channel protein | 492-516 | [27] |
|  | TPSYVAFTDTERLIGDA | HSP 70 | 38-54 | [27] |
|  | TPSYVAFTDTERLIG | HSP 70 | 38-52 | [27] |
|  | VPGLYSPCRAFFNKEELL | EBV major capsid protein | 1264-1282 | [27] |
|  | VPGLYSPCRAFFNK | EBV major capsid protein | 1264-1277 | [27] |
|  | KVDLTFSKQHALLCSDYQADYES | Bovine apolipoprotein B-100 | 1586-1608 | [27] |
|  | KVDLTFSKQHALLCS | Bovine apolipoprotein B-100 | 1586-1600 | [27] |
|  | FSHDYRGSTSHRL | Bovine apolipoprotein B-100 | 1942-1954 | [27] |
|  | LPKYFEKKRNTII | Bovine apolipoprotein B-100 | 2077-2089 | [27] |
|  | APVLISQKLSPIYNLVPVK | Bovine complement C9 | 465-483 | [27] |
|  | TSILCYRKREWIK | Leukaemia inhibitory factor receptor | 854-866 | [27] |
|  | PAFRFTREAAQDCEV | Thromboxane-A synthase | 406-420 | [27] |
|  |  |  |  |  |
| DRB1*03:01 | VDTFLEDVKNLYHSEA | αı-antitrypsin | 149-164 | [28] |
|  | YPNFIMDPKELDKV | Endothelin-converting enzyme 1 | 506-519 | [28] |
|  | KQTISPDYRNMI | IgG2a |  | [28] |
|  | ISNQLTLDSNTKYFHKLN | Bovine apolipoprotein B | 2877-94 | [28] |
|  | KPRAIVVDPVHGFMY | LDL receptor | 518-532 | [28] |
|  | NIQLINDQEVARFD | Uncharacterized |  | [28] |
|  | PPEVTVLTNSPVELREPNV | HLA-DR α chain | 111-129 | [29] |
|  | PPEVTVLTNSPVELREPN | HLA-DR α chain | 111-128 | [29] |
|  | ATKYGNMTEDHVMHLLQNA | Invariant chain | 115-133 | [29] |
|  | VFLLLLADKVPETSLS | Acetylcholine receptor | 289-304 | [29] |
|  | YGYTSYDTFSWAFL | Na+ channel protein | 384-397 | [29] |
|  | GQVKKNNHQEDKIE | CD45 | 1071-1084 | [29] |
|  | LNKILLDEQAQWK | ICAM-2 | 64-76 | [29] |
|  | GPPKLDIRKEEKQIMIDIFHP | IFNγ receptor | 128-148 | [29] |
|  | GPPKLDIRKEEKQIMIDIFH | IFNγ receptor | 128-147 | [29] |
|  | KELKRQYEKKLRQ | EBV tegument p140 | 1395-1407 | [29] |
|  | SPLQALDFFGNGPPVNYKTGNL | IP-30 | 38-59 | [29] |
|  | SPLQALDFFGNGPPVNYKTG | IP-30 | 38-57 | [29] |
|  | GKFAIRPDKKSNPIIRTV | NADH-cytochrome b5 reductase | 155-172 | [29] |
|  | IPDNLFLKSDGRIKYTLNKN | Bovine apolipoprotein B-100 | 1273-1292 | [29] |
|  | TFDEIASGFRQGGASQ | Glucose transporter | 459-474 | [29] |
|  | TGHGARTSTEPTTDY | EBV gp220 | 592-606 | [29] |
|  | LPKPPKPVSKMRMATPLLMQALPM | Invariant chain | 81-104 | [29] |
|  |  |  |  |  |
| DRB1*01:01 | STPEFTILNTFHIPSFTI | Apolipoprotein B | 2646-2663 | [30] |
|  | LDHKFDLMYAKRAFVHWY | Tubulin α 1 chain | 391-408 | [30] |
|  | YKHTLNQIDSVKVWPRRPT | Bovine fetuin | 56-74 | [29] |
|  | YKHTLNQIDSVKVWPRRP | Bovine fetuin | 56-73 | [29] |
|  | LPKPPKPVSKMRMATPLLMQALPMG | Invariant chain | 81-105 | [29] |
|  | LPKPPKPVSKMRMATPLLMQALPM | Invariant chain | 81-104 | [29] |
|  | LPKPPKPVSKMRMATPLLMQALP | Invariant chain | 81-103 | [29] |
|  | LPKPPKPVSKMRMATPLLMQAL | Invariant chain | 81-102 | [30] |
|  | PKPPKPVSKMRMATPLLMQALPMG | Invariant chain | 82-105 | [29] |
|  | PKPPKPVSKMRMATPLLMQALPM | Invariant chain | 82-104 | [29] |
|  | PKPPKPVSKMRMATPLLMQALP | Invariant chain | 82-103 | [29] |
|  | KPPKPVSKMRMATPLLMQALPM | Invariant chain | 83-104 | [29] |
|  | KPPKPVSKMRMATPLLMQALP | Invariant chain | 83-103 | [29] |
|  | PPKPVSKMRMATPLLMQALP | Invariant chain | 84-103 | [29] |
|  | KMRMATPLLMQALPM | Invariant chain | 90-104 | [29] |
|  | KMRMATPLLMQALP | Invariant chain | 90-103 | [29] |
|  | VGSDWRFLRGYHQYAYDG | HLA-A2 | 103-120 | [29] |
|  | VGSDWRFLRGYHQYA | HLA-A2 | 103-117 | [29] |
|  | VGSDWRFLRGYHQY | HLA-A2 | 103-116 | [29] |
|  | GSDWRFLRGYHQYA | HLA-A2 | 104-117 | [29] |
|  | SDWRFLRGYHQYA | HLA-A2 | 105-117 | [29] |
|  | IPADLRIISANGCKVDNS | (Na+/K+) ATPase | 199-216 | [29] |
|  | RVEYHFLSPYVSPKESP | Transferrin receptor | 680-696 | [29] |
|  | LATWTIQGAANALSGDVW | Transferrin receptor | 737-754 | [30] |
|  | HPNQPFYILKPQMPWELW | Sialyltransferase | 288-305 | [30] |
|  | AILEFRAMAQFSRKTD | Uncharacterized |  | [31] |
|  |  |  |  |  |
| DRB1*11:01 | ERPTYTNLNRLIGQIVSS | Tubulin α chain | 220-237 | [30] |
|  | DLHSYVVMNHGRSYTAIS | Nidogen | 429-446 | [30] |
|  | IGRYYTVFDRDNNRVGFA | Cathepsin D | 221-238 | [30] |
|  | VPYRYLQRRKKKGKADGG | Membrane cofactor protein | 315-332 | [30] |
|  | SGRFFTVKLPVALDPGAK | Ribophorin I | 86-103 | [30] |
|  | LPFFIVALVLPFCESSCH | Serotonin receptor | 359-376 | [30] |
|  | CPAGYTCNVKARSCEKEV | Granulin D | 41-58 | [30] |
|  | VGSDWRFLRGYHQYAYDG | HLA-A2 | 103-120 | [30] |
|  | TPTLVEVSRSLGKVGTRC | Bovine serum albumin | 419-436 | [30] |
|  | TTYKKVVFRKYLDSTFTK | Coagulation factor V | 39-56 | [30] |
|  |  |  |  |  |
| DRB1*13:01 | TPKIQVYSRHPAENGKSN | β2-Microglobulin | 21-38 | [30] |
|  | IDSVKVWPRRPTGEVYDI | Bovine fetuin | 45-62 | [30] |
|  | TERVRLVTRHIYNREEYV | HLA-DQB1*0604 | 53-70 | [30] |
|  | SPEFILYAR | Uncharacterized |  | [30] |
|  | DAVLRFNGAPTANFQQDV | Sialyltransferase | 206-223 | [30] |
|  | CPEKWINFQRKCYYFGKG | Low-affinity IgE receptor | 163-180 | [30] |
|  | FYPGQIKVRWFRNDQEET | HLA-DQB1*0604 | 123-140 | [30] |
|  | TERVRLVTRHIYNREE | HLA-DQB1*0603 | 21-36 | [32] |
|  | TERVRLVTRHIYNRE | HLA-DQB1*0603 | 21-35 | [32] |
|  | TERVRLVTRHIYNR | HLA-DQB1*0603 | 21-34 | [32] |
|  | TPKIQVYSRHPAENGKS | β2-Microglobulin | 4-20 | [32] |
|  | TPKIQVYSRHPAENGK | β2-Microglobulin | 4-19 | [32] |
|  | TPKIQVYSRHPAENG | β2-Microglobulin | 4-18 | [32] |
|  | TPKIQVYSRHPAEN | β2-Microglobulin | 4-17 | [32] |
|  | GPDGRLLRGHDQYAYDGKDY | HLA-B7 | 104-123 | [32] |
|  | GPDGRLLRGHDQYAYDGKD | HLA-B7 | 104-122 | [32] |
|  | LPKPPKPVSKMRMATPLLMQALPM | Invariant chain | 81-104 | [32] |
|  | LPKPPKPVSKMRMATPLLMQALP | Invariant chain | 81-103 | [32] |

Supplementary material

References

1. Nowak, J., Mika-Witkowska, R., Polak, et al. (2008). Allele and extended haplotype polymorphism of HLA-A, -C, -B, -DRB1 and -DQB1 loci in Polish population and genetic affinities to other populations. *Tissue Antigens. 71*, 193-205.

2. Sudo, T., Kamikawaji, N., Kimura, A., et al. (1995). Differences in MHC class I self peptide repertoires among HLA-A2 subtypes. *Journal of immunology. 155*, 4749-4756.

3. Henderson, R.A., Michel, H., Sakaguchi, K., et al. (1992). HLA-A2.1-associated peptides from a mutant cell line: a second pathway of antigen presentation. *Science. 255*, 1264-1266.

4. Wei, M.L., & Cresswell, P. (1992.) HLA-A2 molecules in an antigen-processing mutant cell contain signal sequence-derived peptides. *Nature. 356*, 443-446.

5. Hunt, D.F., Henderson, R.A., Shabanowitz, J., et al. (1992). Characterization of peptides bound to the class I MHC molecule HLA-A2.1 by mass spectrometry. *Science. 255*, 1261-1263.

6. Papadopoulos, K.P., Suciu-Foca, N., Hesdorffer, C.S., et al. (1997). Naturally processed tissue- and differentiation stage-specific autologous peptides bound by HLA class I and II molecules of chronic myeloid leukemia blasts. *Blood. 90*, 4938-4946.

7. Kubo, R.T., Sette, A., Grey, H.M., et al. (1994). Definition of specific peptide motifs for four major HLA-A alleles. *Journal of Immunology. 152*, 3913-3924.

8. DiBrino, M., Parker, K.C., Shiloach, J., et al. (1994). Endogenous peptides with distinct amino acid anchor residue motifs bind to HLA-A1 and HLA-B8. *Journal of Immunology. 152*, 620-631.

9. Falk, K., Rotzschke, O., Takiguchi, M., (1994). Peptide motifs of HLA-A1, -A11, -A31, and -A33 molecules. *Immunogenetics. 40*, 238-241.

10. van Baalen, C.A., Klein, M.R., Huisman, R.C., et al. (1996). Fine-specificity of cytotoxic T lymphocytes which recognize conserved epitopes of the Gag protein of human immunodeficiency virus type 1. *Journal of General Virology. 77*, 1659-1665.

11. Rickinson, A.B., & Moss, D.J. (1997). Human cytotoxic T lymphocyte responses to Epstein-Barr virus infection. *Annual Review of Immunology. 15*, 405-431.

12. Barber, L.D., Gillece-Castro, B., Percival, L., et al. (1995). Overlap in the repertoires of peptides bound in vivo by a group of related class I HLA-B allotypes. Current Biology. 5, 179-190.

13. Huczko, E.L., Bodnar, W.M., Benjamin, D., et al. (1993). Characteristics of endogenous peptides eluted from the class I MHC molecule HLA-B7 determined by mass spectrometry and computer modeling. Journal of Immunology. 151, 2572-2587.

14. Engelhard, V.H. (1994). Structure of peptides associated with class I and class II MHC molecules. *Annual Review of Immunology.* 12, 181-207.

15. Falk, K., Rotzschke, O., Takiguchi, M., et al. (1995). Peptide motifs of HLA-B51, -B52 and -B78 molecules, and implications for Behcet's disease. *International Immunology. 7*, 223-228.

16. Falk, K., Rotzschke, O., Takiguchi, M., et al. (1995). Peptide motifs of HLA-B58, B60, B61, and B62 molecules. *Immunogenetics. 41*, 165-168.

17. Barber, L.D., Percival, L., Valiante, N.M., et al. (1996). The inter-locus recombinant HLA-B*4601 has high selectivity in peptide binding and functions characteristic of HLA-C. *Journal of Experimental Medicine. 184*, 735-740.

18. Prilliman, K.R., Lindsey, M., Jackson, K.W., et al. (1998). Complexity among constituents of the HLA-B*1501 peptide motif. *Immunogenetics. 48*, 89-97.

19. Rotzschke, O., Falk, K., Stevanovic, S., et al. (1994). Dominant aromatic/aliphatic C-terminal anchor in HLA-B*2702 and B*2705 peptide motifs. *Immunogenetics. 39*, 74-77.

20. Jardetzky, T.S., Lane, W.S., Robinson, R.A., Madden, D.R., & Wiley, D.C. (1991). Identification of self peptides bound to purified HLA-B27. *Nature. 353*, 326-329.

21. Fiorillo, M.T., Meadows, L., D'Amato, M., et al. (1997). Susceptibility to ankylosing spondylitis correlates with the C-terminal residue of peptides presented by various HLA-B27 subtypes. *European Journal of Immunology. 27*, 368-373.

22. Boisgerault, F., Tieng, V., Stolzenberg, M.C., et al. (1996). Differences in endogenous peptides presented by HLA-B*2705 and B*2703 allelic variants. Implications for susceptibility to spondylarthropathies. *Journal of Clinical Investigation. 98*, 2764-2770.

23. Frumento, G., Harris, P.E., Gawinowicz, M.A., Suciu-Foca, N., & Pernis, B. (1993). Sequence of a prominent 16-residue self-peptide bound to HLA-B27 in a lymphoblastoid cell line. *Cellular Immunology. 152*, 623-626.

24. Takamiya, Y., Schonbach, C., Nokihara, K., et al. (1994). HLA-B*3501-peptide interactions: role of anchor residues of peptides in their binding to HLA-B*3501 molecules. *International Immunology. 6*, 255-261.

25. DiBrino, M., Parker, K.C., Margulies, D.H., et al. (1995). Identification of the peptide binding motif for HLA-B44, one of the most common HLA-B alleles in the Caucasian population. *Biochemistry. 34*, 10130-10138.

26. Fleischhauer, K., Avila, D., Vilbois, F., et al. (1994). Characterization of natural peptide ligands for HLA-B*4402 and -B*4403: implications for peptide involvement in allorecognition of a single amino acid change in the HLA-B44 heavy chain. *Tissue Antigens. 44*, 311-317.

27. Chicz, R.M., Urban, R.G., Gorga, J.C., et al. (1993). Specificity and promiscuity among naturally processed peptides bound to HLA-DR alleles. *Journal of Experimental Medicine. 178*, 27-47.

28. Malcherek, G., Falk, K., Rotzschke, O., et al. (1993). Natural peptide ligand motifs of two HLA molecules associated with myasthenia gravis. *International Immunology. 5*, 1229-1237.

29. Chicz, R.M., Urban, R.G., Lane, W.S., et al. (1992). Predominant naturally processed peptides bound to HLA-DR1 are derived from MHC-related molecules and are heterogeneous in size. *Nature. 358,* 764-768.

30. Verreck, F.A., van de Poel, A., Drijfhout, J.W., et al. (1996). Natural peptides isolated from Gly86/Val86-containing variants of HLA-DR1, -DR11, -DR13, and -DR52. *Immunogenetics. 43*, 392-397.

31. Kropshofer, H., Max, H., Muller, C.A., et al. (1992). Self-peptide released from class II HLA-DR1 exhibits a hydrophobic two-residue contact motif. Journal of Experimental *Medicine. 175*, 1799-1803.

32. Davenport, M.P., Quinn, C.L., Chicz, R.M., et al. (1995). Naturally processed peptides from two disease-resistance-associated HLA-DR13 alleles show related sequence motifs and the effects of the dimorphism at position 86 of the HLA-DR beta chain. *Proceedings of National Academy of Science USA. 92*, 6567-6571.
